# Effects of various degrees of esterification on antioxidant and immunostimulatory activities of okra pectic-polysaccharides

**DOI:** 10.3389/fnut.2022.1025897

**Published:** 2022-10-20

**Authors:** Wei Li, Jie Li, Jin Wang, Yuan He, Yi-Chen Hu, Ding-Tao Wu, Liang Zou

**Affiliations:** ^1^Key Laboratory of Coarse Cereal Processing (Ministry of Agriculture and Rural Affairs), Sichuan Engineering and Technology Research Center of Coarse Cereal Industrialization, School of Food and Biological Engineering, Chengdu University, Chengdu, Sichuan, China; ^2^School of Preclinical Medicine, Chengdu University, Chengdu, Sichuan, China; ^3^Sichuan Institute of Food Inspection, Chengdu, Sichuan, China

**Keywords:** *Abelmoschus esculentus*, pectic-polysaccharides, structure-activity relationship, degree of esterification, immunostimulatory effect

## Abstract

Pectic-polysaccharides are considered as one of the most abundant bioactive components in okra, which possess various promising health-promoting effects. However, the knowledge regarding the structure-bioactivity relationship of okra pectic-polysaccharides (OPP) is still limited. In this study, effects of various degrees of esterification (DEs) on *in vitro* antioxidant and immunostimulatory activities of OPP were analyzed. Results displayed that OPP with high (42.13%), middle (25.88%), and low (4.77%) DE values were successfully prepared by mild alkaline de-esterification, and their primary chemical structures (compositional monosaccharide and glycosidic linkage) and molecular characteristics (molecular weight distribution, particle size, and rheological property) were overall stable. Additionally, results showed that the notable decrease of DE value did not significantly affect antioxidant activities [2,2’-azino-bis (3-ethylbenzthiazoline-6-sulphonic acid) (ABTS) and nitric oxide (NO) radical scavenging abilities as well as ferric reducing antioxidant power (FRAP)] of OPP, suggesting that the DE was not closely related to its antioxidant activity. In fact, the slight decrease of antioxidant activity of OPP after the alkaline de-esterification might be attributed to the slight decrease of uronic acid content. Nevertheless, the immunostimulatory effect of OPP was closely related to its DE, and a suitable degree of acetylation was beneficial to its *in vitro* immunostimulatory effect. Besides, the complete de-acetylation resulted in a remarkable reduction of immune response. The findings are beneficial to better understanding the effect of DE value on antioxidant and immunomodulatory activities of OPP, which also provide theoretical foundations for developing OPP as functional foods or health products.

## Introduction

Pectic-polysaccharides are complex heteropolysaccharides existed in the primary cell walls of vegetables and fruits, which are predominantly composed of homogalacturonan (HG), rhamnogalacturonan I (RG-I), and rhamnogalacturonan II (RG-II) domains (1). Recently, pectic-polysaccharides extracted from vegetables and fruits have attracted increasing attention to be developed into functional food ingredients owing to their various health-promoting properties, such as antioxidant, anti-inflammatory, immunomodulatory, anti-tumor, anti-hyperlipidemic, anti-hyperglycemic, anti-obesity, and prebiotic properties (1–3). A great number of studies have demonstrated that the ratio of HG/RG-I, molecular mass, branched chain length, degree of esterification (DE), glycosidic linkage, and compositional monosaccharide of pectic-polysaccharides are critical chemical structures for their health beneficial effects (1–3). Nevertheless, the knowledge regarding the precise structure-biological activity relationships of pectic-polysaccharides is still limited because of a lack of pure samples and fine structure analysis. Therefore, it is important to uncover the relationship between the precise structures of pectic-polysaccharides and their biological activities, which is beneficial to better promoting the application of pectic-polysaccharides in the functional food industry.

*Abelmoschus esculentus* L. Moench, known as okra or lady’s finger, is a vital edible and medicinal plant in China. It is native to the Africa but can now be found throughout tropical and subtropical areas of the world (4). Okra is not only consumed as a delicious vegetable, but also utilized as a folk medicine for the treatment of various diseases (5). Due to its promising health benefits, such as antioxidant, immunomodulatory, anti-diabetic, anti-cancer, anti-hypertensive, and anti-microbial effects, okra has attracted increasing attention to be developed and utilized as functional foods in recent years (4). Lots of studies have demonstrated that pectic-polysaccharides, proteins, flavonoids, and phenolic acids exist as the major bioactive components in okra, which contribute to its various beneficial properties (4, 6). Especially, pectic-polysaccharides are considered as one of the most abundant bioactive components in okra, which play a critical role in its biological activities (6). Indeed, the backbone of okra pectic-polysaccharides (OPP) is identified as →4)-α-D-GalA*p*-(1→2,4)-α-L-Rha*p*-(1→, confirming that the RG I segment is rich in okra (7–9). Besides, the HG segment is also found in okra (6). Indeed, OPP also have a low degree of methyl esterification and a high degree of acetylation (10, 11). Generally, pectic-polysaccharides are complex biomacromolecules, their physicochemical or structural features can directly impact the biological functions. Several studies have indicated that the molecular mass of OPP significantly affect their antioxidant, prebiotic, and anti-inflammatory activities (12, 13), as well as immune stimulating activity (14). Besides, a recent study has shown that biological activities of OPP can be improved through the degradation by ultrasound assisted H_2_O_2_/Vc reaction, and the *in vitro* antioxidant and immunostimulatory effects of OPP are related to its molecular mass, branched chain length, and DE (9). Furthermore, the DE value has gained much attention in the investigation of pectic-polysaccharides, because the DE value can obviously affect biological activities and functional properties of pectic-polysaccharides, such as inhibitory effect on α-amylase, modulation of gut microbial composition, immunoregulatory effect, gel property, and emulsifying ability (15–18). Indeed, the mild alkaline de-esterification has been considered as one of the most important methods to reduce the esterification of pectic-polysaccharides (15, 19). However, the potential relationships between DE value and biological activity of OPPare still unclear, which require to be systematically investigated.

Therefore, in order to further clarify the potential structure-bioactivity relationship of OPP, effects of various degrees of esterification on *in vitro* antioxidant capacities and immunostimulatory activities of OPP were investigated in the present study.

## Materials and methods

### Materials and chemicals

Okra fruits of *Abelmoschus esculentus* cv. Wufu used in this study were harvested from Chengdu, Sichuan Province, China. Monosaccharide standards, 2,2′-azino-bis (3-ethylbenzthiazoline-6-sulphonic acid) (ABTS), sodium nitroprusside (SNP), vitamin C (Vc), griess reagent, lipopolysaccharide (LPS), and 3-(4,5-dimethylthiazol-2-yl)-2,5-diphenyl tetrazolium bromide (MTT) were purchased from Sigma-Aldrich (St. Louis, MO, USA).

### Preparation of okra pectic-polysaccharides with various degrees of esterification

The preparation of purified OPP was performed as previously reported (20). Briefly, the crude water-soluble polysaccharides from okra fruit powders were extracted by ultrasound assisted-extraction (650 W, 24 kHz, Scientz, Ningbo, China) as previously reported (20). Afterward, the supernatants were sequentially precipitated (three volumes of 95% ethanol), redissolved, dialyzed (molecular mass cutoff, 3.5 kDa), and separated by a DEAE anion exchange column (5 × 50 cm) to prepare purified OPP. Moreover, the modification of OPP was carried out to improve its *in vitro* biological activities as previously reported (9). Briefly, 50.0 mL of OPP solutions (10.0 mg/mL) were mixed with ascorbic acid and H_2_O_2_ at the final concentrations of 20.0 and 40.0 mM, respectively, and then degraded by ultrasound (650 W, 24 kHz, Scientz, Ningbo, China) at the power of 520 W for 0.5 h (9). Finally, the degraded product of okra pectic-polysaccharides (DOPP) with promoted biological functions was obtained.

Furthermore, the preparation of DOPP with various DE values was carried out according to a previous study with a few modifications (19). In brief, the mild alkaline de-esterification was carried out by stirring 0.5% (*w/v*) of DOPP in NaOH solution at basic pH values of 11.0 and 13.0 for 0.5 h under 4°C, respectively. At the end of reaction, the sample solution was acidified to pH = 6.0 by adding HCl (1 M). Then, after dialysis (molecular mass cutoff, 3.5 kDa) and freeze-drying in turn, okra pectic-polysaccharides with a middle DE value (DOPP-MDE) and a low DE value (DOPP-LDE) were obtained. Indeed, the yields of DOPP-MDE and DOPP-LDE were measured to be 95.73 and 90.82%, respectively. Correspondingly, the original DOPP was named DOPP-HDE, which possessed a relatively high DE value.

### Structural characterization of okra pectic-polysaccharides with various degrees of esterification

Total polysaccharides, uronic acids, and proteins of OPP with various DE values were detected by colorimetric methods as previously reported (21). Molecular weight (*M*_*w*_), molecular weight distribution (*M_*w*_/M_*n*_*), and radius of gyration (*R*_*g*_) as well as rheological property of DOPP-HDE, DOPP-MDE, and DOPP-LDE were also measured as previously reported (9, 22). In brief, a TSKgel GMPWXL column (300 × 7.8 mm, i.d.) was utilized for the separation of DOPP-HDE, DOPP-MDE, and DOPP-LDE, respectively. Both multi-angle laser light scattering detection and refractive index detection (Wyatt Technology Co., Santa Barbara, CA, USA) were applied for the analysis of DOPP-HDE, DOPP-MDE, and DOPP-LDE, respectively. The apparent viscosities of DOPP-HDE, DOPP-MDE, and DOPP-LDE were measured by a Discovery Hybrid Rheometer-1 (DHR-1, TA Instruments, New Castle, DE, USA). For the investigation of primary chemical structures, the monosaccharide compositions, FT-IR spectra, and ^1^H NMR spectra of OPP with various DE values were analyzed. In brief, monosaccharide compositions of DOPP-HDE, DOPP-MDE, and DOPP-LDE were analyzed by HPLC (Thermo Fisher Scientific, Waltham, MA, USA) as previously reported (23). A C18 column (150 × 4.6 mm, 5 μm, Thermo Fisher Scientific, Waltham, MA, USA) was carried out for the separation of monosaccharides, and the signals were recorded at 245 nm. Additionally, ^1^H NMR spectra of DOPP-HDE, DOPP-MDE, and DOPP-LDE were also recorded on a Bruker Ascend 600 MHz spectrometer (Bruker, Rheinstetten, Germany) as previously reported (24, 25). Furthermore, the FT-IR spectra of DOPP-HDE, DOPP-MDE, and DOPP-LDE were also analyzed according to a previous reported method (26, 27). Indeed, the DE value was estimated based on the FT-IR spectra at 1,700–1,750 cm^–1^ (about 1,730 cm^–1^) and 1,600–1,640 cm^–1^ (about 1,635 cm^–1^), which was estimated based on the following equation:


$$\mathrm{DE}(\%)=\left(\frac{\mathrm{A1730}}{\mathrm{A1730}+\mathrm{A1635}}\right)\times 100$$

### Evaluation of *in vitro* antioxidant activities of okra pectic-polysaccharides with various degrees of esterification

The ferric reducing antioxidant power (FRAP), ABTS radical scavenging ability, and nitric oxide (NO) radical scavenging ability of OPP with various DE values were evaluated according to previously reported methods (26). In brief, for the determination of ABTS radical scavenging ability, the ABTS radical cation working solution (200 μL) was mixed with 20 μL of each sample (2.0–10.0 mg/mL) in a 96-well microplate to react at 30°C for 20 min; for the determination of NO radical scavenging ability, each sample (450 μL, 2.0–10.0 mg/mL) was mixed with 50 μL of SNP (10 mM) to react at 25°C for 3 h, and then 250 μL of Griess reagent was added. Besides, the IC_50_ values (mg/mL) of DOPP-HDE, DOPP-MDE, and DOPP-LDE for scavenging free radicals could be determined on the basis of a logarithmic regression curve. Additionally, for the determination of FRAP, 100 μL of each sample (2.0–10.0 mg/mL) was mixed with 100 μL of potassium ferricyanide (1%, *w/w*) at 50°C for 20 min, and then 100 μL of trichloroacetic acid (10%, *w/v*) was added and centrifugated. Finally, both distilled water (100 μL) and ferric chloride (20 μL) were added into the supernatant (100 μL). The absorbance of the mixture was recorded at 593 nm. Vc was used as a positive control in each experiment.

### Evaluation of immunostimulatory activities of okra pectic-polysaccharides with various degrees of esterification

Immunostimulatory activities of OPP with various DE values were evaluated by using an *in vitro* model of RAW 264.7 macrophages according to a previously reported method (9). In brief, effects of OPP with various DEs at the concentrations ranged from 5 to 320 μg/mL on the proliferation of RAW 264.7 macrophages were determined by the MTT colorimetric method. Additionally, the production of NO and release of cytokines [interleukin-6 (IL-6) and tumor necrosis factor-α (TNF-α)] from RAW 264.7 macrophages were also detected as previously reported (9). After the RAW 264.7 macrophage was stimulated with DOPP-HDE, DOPP-MDE, and DOPP-LDE at the concentrations ranged from 5 to 320 μg/mL, the NO production was measured by Griess reagent. Besides, the release of IL-6 and TNF-α from RAW 264.7 macrophages were measured by ELISA kits according to the manufacturer’s procedures (eBioscience, San Diego, CA, USA).

### Statistical analysis

Each experiment was carried out in triplicate. Data are presented as mean ± standard deviation. Statistical analysis was performed by using a two-tailed Student’s *t*-test and one-way analysis of variance followed by a Duncan’s test, respectively.

## Results and discussion

### Structural features of okra pectic-polysaccharides with various degrees of esterification

#### Chemical structures of okra pectic-polysaccharides with various degrees of esterification

The chemical compositions of OPP with different DE values are summarized in Table 1. Total polysaccharides in DOPP-HDE, DOPP-MDE, and DOPP-LDE were detected to be 92.59, 92.26, and 93.21%, respectively, indicating that the contents of total polysaccharides were not affected by mild alkaline de-esterification. However, total uronic acids in DOPP-HDE, DOPP-MDE, and DOPP-LDE slightly decreased from 33.51 to 27.51% by mild alkaline de-esterification, which might be due to the fact that the elimination reaction could induce the hydrolysis of pectic-polysaccharides by splitting their backbone (15, 28). Additionally, minor proteins were found in DOPP-HDE, DOPP-MDE, and DOPP-LDE, which were similar with a previous study (9).

**TABLE 1:** Chemical composition, molecular weight (*M*_*w*_), polydispersity (*M_*w*_/M_*n*_*), radius of gyration (*R*_*g*_), and constituent monosaccharide of okra pectic-polysaccharides (OPP) with various degrees of esterification.

	DOPP-HDE	DOPP-MDE	DOPP-LDE
Total polysaccharides (%)	92.59 ± 2.31^a^	92.26 ± 3.16^a^	93.21 ± 2.00^a^
Total uronic acids (%)	33.51 ± 1.64^a^	32.91 ± 1.93^a^	27.51 ± 1.77^b^
Total proteins (%)	1.25 ± 0.27^a^	1.00 ± 0.04^a^	0.81 ± 0.32^a^
Esterification degree (%)	42.13 ± 0.14^a^	25.88 ± 0.47^b^	4.77 ± 0.34^c^
*M_*w*_* × 10^5^ (Da)	1.897 (±0.928%)^a^	1.865 (±0.693%)^a^	1.753 (±1.036%)^a^
*M_*w*_/M_*n*_*	1.786 (±1.381%)	1.736 (±1.049%)	1.773 (±1.658%)
*R*_*g*_	30.3 (±2.9%)^a^	30.0 (±3.6%)^a^	29.6 (±2.4%)^a^
Monosaccharide compositions and molar ratios			
Rhamnose	1	1	1
Galactose	2.14	2.11	2.08
Galacturonic acid	1.10	1.09	1.07
Mannose	0.48	0.37	0.34
Arabinose	0.19	0.17	0.16
Glucuronic acid	Trace	Trace	Trace

DOPP-HDE, DOPP-MDE, and DOPP-LDE indicate okra pectic-polysaccharides with high, middle, and low degrees of esterification, respectively; total polysaccharides (%, *w*/*w*), uronic acids (%, *w*/*w*), and proteins (%, *w*/*w*) indicate the total content of neutral and acidic polysaccharides, the total content of uronic acids, and the total content of proteins in okra pectic-polysaccharides; esterification degree (%) indicates the esterification degree of total uronic acids; different superscript lowercase letters indicated significance (*p* < 0.05) in each row.

**FIGURE 1:**
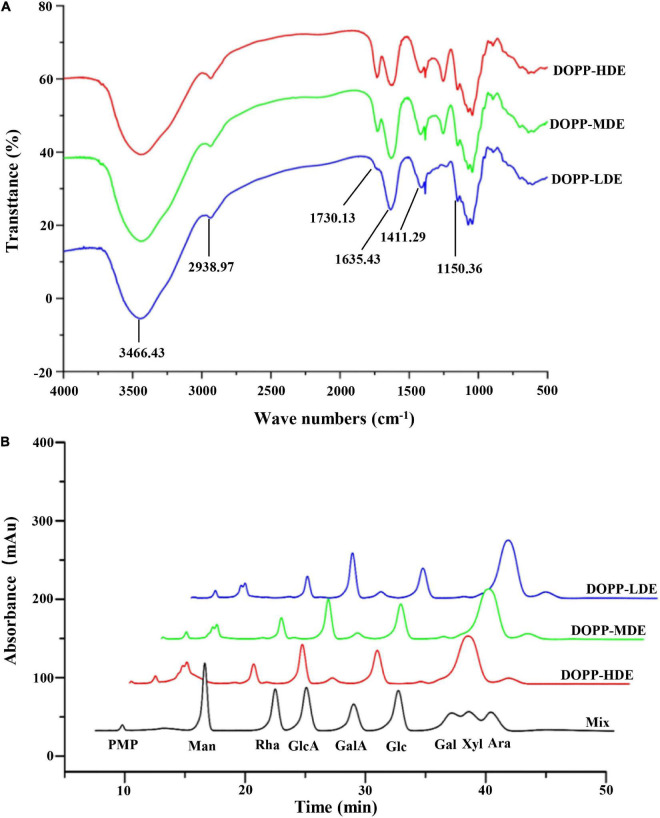
FT-IR spectra **(A)** and high-performance liquid chromatograms of compositional monosaccharides **(B)** of okra pectic-polysaccharides (OPP) with various degrees of esterification. DOPP-HDE, DOPP-MDE, and DOPP-LDE indicate OPP with high, middle, and low degrees of esterification, respectively; Mix indicates the monosaccharide standards, which was analyzed by HPLC under the same conditions of samples. PMP, 1-phenyl-3-methyl-5-pyrazolone; Man, mannose; Rha, rhamnose; GlcA, glucuronic acid; GalA, galacturonic acid; Glc, glucose; Gal, galactose; Xyl, xylose; Ara, arabinose.

**FIGURE 2:**
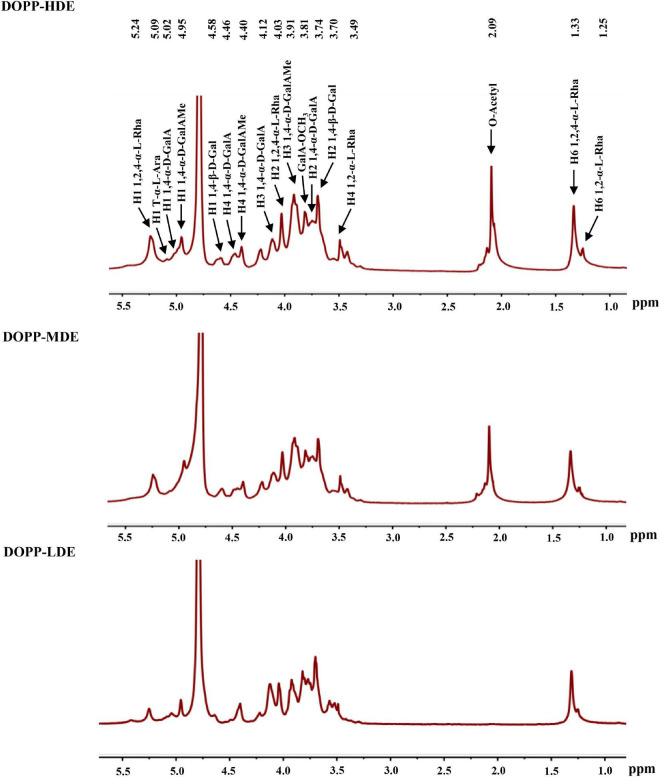
^1^H NMR spectra of okra pectic-polysaccharides (OPP) with various degrees of esterification. The sample codes were the same in Figure 1.

Furthermore, in order to confirm the primary chemical structures of OPP with different DE values, the monosaccharide compositions, FT-IR spectra, and ^1^H NMR spectra were systematically analyzed. As shown in Figure 1A, similar FT-IR spectra were found in DOPP-HDE, DOPP-MDE, and DOPP-LDE, indicating that the major chemical groups of OPP were stable after the treatment of mild alkaline de-esterification. The typical absorption bands of pectic-polysaccharides, including 3466.43, 2938.97, 1730.13, 1635.43, 1411.29, and 1150.36 cm^–1^, were found in all tested samples (28, 29). However, as shown in Figure 1A, the peak areas of absorption band at around 1730.13 cm^–1^ related to esterified functional groups remarkably changed after the treatment of mild alkaline de-esterification (28). Indeed, the DE values of DOPP-HDE, DOPP-MDE, and DOPP-LDE were estimated to be 42.13, 25.88, and 4.77% based on the peak areas of absorption bands at around 1730.13 and 1635.43 cm^–1^ (Table 1), respectively, indicating that OPP with various DE values were successfully prepared. Additionally, as shown in Figure 1B, the same types of monosaccharides were found in DOPP-HDE, DOPP-MDE, and DOPP-LDE, and galacturonic acid, rhamnose, and galactose were determined as the major monosaccharides as previously reported (9). Indeed, similar molar ratios of constituent monosaccharides were also found in all samples (Table 1), suggesting that the primary chemical structures of OPP, except the DE, were relatively stable after the treatment of mild alkaline de-esterification. Furthermore, ^1^H NMR spectra of OPP with various DE values were also analyzed for the confirmation of their chemical structures (Figure 2). More specifically, the signal at around 2.09 ppm in DOPP-HDE was assigned to acetyl groups (10, 11), which might locate on *O*-2 or *O*-3 of galacturonosyl residues and *O*-3 of rhamnosyl residues (11). The intensity of this signal obviously decreased in DOPP-MDE or even disappeared in DOPP-LDE, indicating that the degree of acetylation of galacturonosyl or rhamnosyl residues in OPP could be decreased by mild alkaline de-esterification. Additionally, the signal at around 3.81 ppm in DOPP-HDE was assigned to methoxyl groups (10, 11). This signal could be also found in DOPP-MDE and DOPP-LDE, suggesting that methoxyl groups bonded to carboxyl groups of galacturonic acid could still exist in OPP under the mild alkaline de-esterification conditions. Similar phenomena were also found in previous studies that the methoxyl group from the esterified units of galacturonic acids could not be completely removed under the mild alkaline conditions (15, 28). Collectively, these results indicated that the decrease of DE value in DOPP-MDE and DOPP-LDE compared to DOPP-HDE might be mainly attributed to the complete de-acetylation and the partial de-methylation.

Moreover, the typical signals, including 1,4-α-D-GalA*p* (H-1, 5.02 ppm), 1,4-α-D-GalAMe*p* (H-1, 4.95 ppm), 1,2-α-L-Rha*p* (H-6, 1.25 ppm), 1,2,4-α-L-Rha*p* (H-1/H-6, 5.24/1.33 ppm), 1,4-β-D-Gal*p* (H-1, 4.58 ppm), could be found in DOPP-HDE, DOPP-MDE, and DOPP-LDE. These results suggested that the RG-I backbone with galactan side chains existed as the main pectic-polysaccharides in DOPP-HDE, DOPP-MDE, and DOPP-LDE (7, 9, 10, 28), and the primary chemical structures of OPP, except the DE, were overall stable after the treatment of mild alkaline de-esterification.

#### Molecular mass and particle size of okra pectic-polysaccharides with various degrees of esterification

**FIGURE 3:**
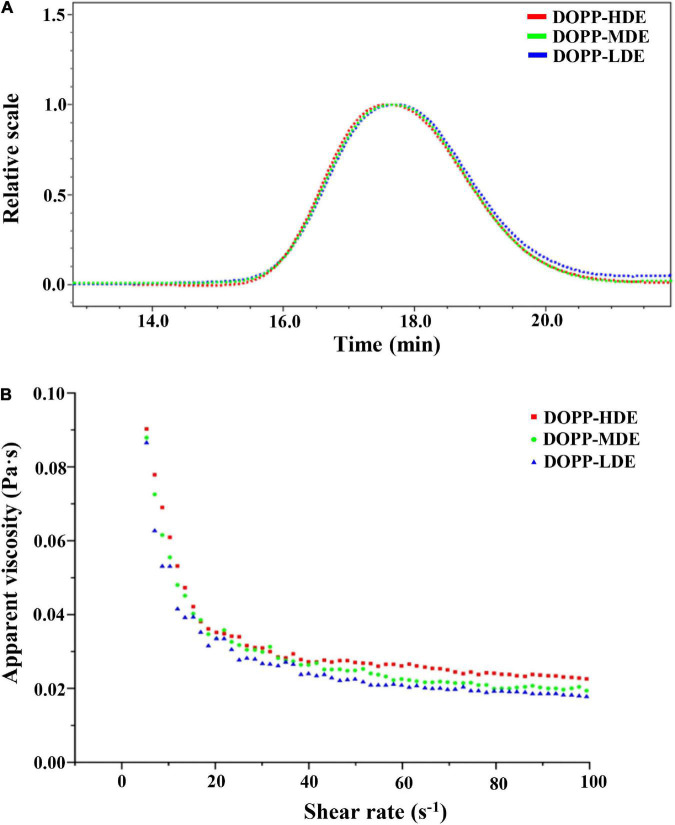
Size exclusion chromatograms **(A)** and dependences of apparent viscosity on the shear rate **(B)** of okra pectic-polysaccharides (OPP) with various degrees of esterification. The sample codes were the same in Figure 1.

Macromolecular characteristics of pectic-polysaccharides, such as molecular mass, molecular mass distribution, and particle size, have significant impacts on their biological properties and applications in the functional food industry (9, 30, 31). Consequently, macromolecular characteristics of OPP with various DE values were measured and compared. As shown in Figure 3A, similar size exclusion chromatography (SEC) profiles were found in OPP with various DE values, which exhibited a single symmetrical peak. Results showed that the retention time of DOPP-HDE, DOPP-MDE, and DOPP-LDE were almost the same, suggesting that the molecular mass and molecular mass distribution of OPP were overall stable after the treatment of mild alkaline de-esterification. Indeed, as shown in Table 1, the molecular masses of DOPP-HDE, DOPP-MDE, and DOPP-LDE were detected to be 1.897 × 10^5^, 1.865 × 10^5^, and 1.753 × 10^5^ Da, respectively, suggesting that the mild alkaline de-esterification could slightly (no significant difference) degrade the molecular weight of OPP. This phenomenon was similar with previous studies that β-elimination reaction could cause the degradation of molecular mass (15, 28). Besides, the molecular mass distributions of OPP with different DE values were similar, which ranged from 1,736 to 1,786. Additionally, corresponding with the changes in molecular mass, the particle size of DOPP-HDE also slightly (no significant difference) decreased from 30.3 to 29.6 nm after the treatment of mild alkaline de-esterification.

#### Rheological properties of okra pectic-polysaccharides with various degrees of esterification

Rheological property is considered as one of the most important factors that affect the biological functions and food applications of pectic-polysaccharides (28, 32). Figure 3B displays the dependences of apparent viscosity on shear rate of OPP (40 mg/mL) with different DE values. As expected, the apparent viscosities of DOPP-HDE, DOPP-MDE, and DOPP-LDE affected by the shear rate. More specifically, the apparent viscosities of each sample declined with the increase of the shear rate ranged from 0.01 to 50 s^–1^, indicating that each sample solution exhibited non-Newtonian shear thinning fluid behavior (32). In addition, the apparent viscosities of each sample declined slightly with the increase of the shear rate ranged from 50 to 100 s^–1^, exhibiting Newtonian flow fluid behavior (9, 21). This rheological character of OPP might be due to the fact that the chains of pectin were arranged in an orderly manner along the fluid direction with the increase of the shear rate, and the interactions between the adjacent chains and the viscosity decreased (33). Furthermore, compared with DOPP-HDE, the apparent viscosities of DOPP-MDE and DOPP-LDE slightly reduced, indicating that the obvious decrease of DE value did not cause a sharp decrease of apparent viscosity. This result is different from a previous study that the pectin with a lower degree of esterification is often accompanied by a decrease of viscosity (34). In this study, DOPP-LDE with a lower degree of esterification also exhibited a higher apparent viscosity which might be attributed to the fact that the viscosity of the sample was affected by several factors, such as molecular mass, chain conformation, monosaccharide composition (9, 21, 32). Collectively, although the DE value of OPP was significantly decreased, its apparent viscosity was relatively stable after the treatment of mild alkaline de-esterification.

### Effects of various degrees of esterification on *in vitro* antioxidant activities of okra pectic-polysaccharides

The antioxidant activity has been demonstrated as one of the most important biological functions of okra (4), and OPP have remarkable *in vitro* antioxidant capacities against different free radicals (9, 21, 26, 35). Several studies have shown that the antioxidant activities of crude OPP may be related to their molecular mass, chain conformation, uronic acid, and DE as well as conjugated polyphenols (21, 26, 35, 36). A recent study also showed that the antioxidant activity of a purified okra pectic-polysaccharide might be closely related to the combination effect of molecular mass and DE (9). However, whether the DE can directly affect the antioxidant activity of OPP is still not clear. Therefore, in the present study, in order to evaluate the precise degree of esterification on *in vitro* antioxidant activity of OPP, three OPP with high, middle, and low degrees of esterification were prepared and their antioxidant activities were evaluated.

**FIGURE 4:**
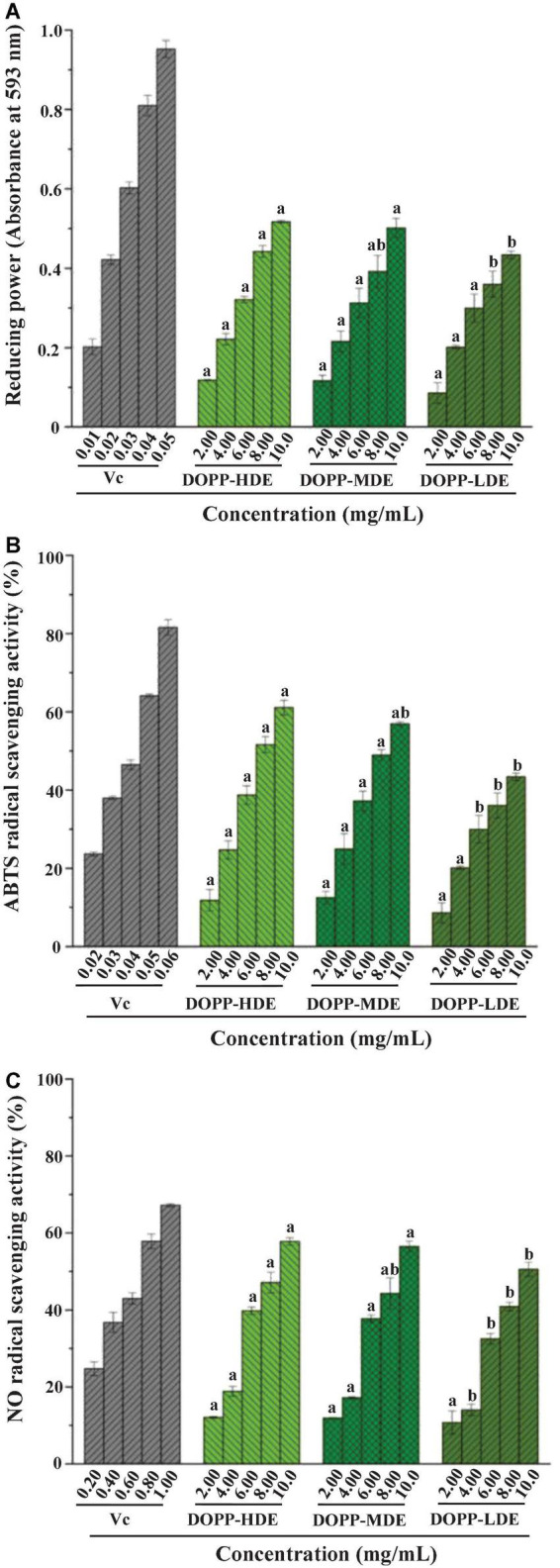
Ferric reducing antioxidant power (FRAP) **(A)**, 2,2′-azino-bis (3-ethylbenzthiazoline-6-sulphonic acid) (ABTS) radical scavenging activity **(B)**, and nitric oxide (NO) radical scavenging activity **(C)** of okra pectic-polysaccharides (OPP) with various degrees of esterification. The sample codes were the same in Figure 1; the error bars are standard deviations; significant (*p* < 0.05) differences among OPP with various degrees of esterification are shown by data bearing different letters (a-b); statistical significances were carried out by ANOVA and Ducan’s test.

Figure 4 displays the FRAPs and ABTS radical scavenging capacities as well as NO radical scavenging capacities of OPP with high, middle, and low DE values. Results showed that OPP with various DE values exhibited remarkable antioxidant activities with a dose-dependent manner. For the FRAP, the absorbance values of DOPP-HDE, DOPP-MDE, and DOPP-LDE at 593 nm were detected to be 0.52 ± 0.01, 0.50 ± 0.02, and 0.43 ± 0.01 at the concentration of 10 mg/mL, respectively, which were lower than that of Vc (0.95 ± 0.02). Additionally, in terms of ABTS radical scavenging activity, the IC_50_ values of DOPP-HDE, DOPP-MDE, and DOPP-LDE were detected to be 7.76 ± 0.31, 8.40 ± 0.32, and 10.24 ± 0.72 mg/mL, respectively, which were higher than that of Vc (0.04 mg/mL). Furthermore, in terms of NO radical scavenging activity, the IC_50_ values of DOPP-HDE, DOPP-MDE, and DOPP-LDE were detected to be 8.43 ± 0.21, 9.02 ± 0.49, and 10.64 ± 0.38 mg/mL, respectively, which were also higher than that of Vc (0.62 ± 0.02 mg/mL). Surprisingly, results showed that DOPP-LDE with the lowest DE value (4.77%) among three samples exhibited the lowest antioxidant activities in the present study. Besides, although the DE value of DOPP-MDE (25.88%) was significantly lower than that of DOPP-HDE (42.13%), the *in vitro* antioxidant activities of DOPP-HDE and DOPP-MDE were similar. Therefore, these results indicated that the remarkable decrease of DE value (mainly degree of acetylation) of OPP by mild alkaline de-esterification did not obviously affect their antioxidant activity, suggesting that the DE was not closely correlated to the antioxidant activity of OPP. This phenomenon is quite different from previous studies that the lower DE of pectic-polysaccharides is closely related to their higher antioxidant activity (37, 38). In fact, the antioxidant activity of pectic-polysaccharides is assigned to their hydrogen-donating abilities, and several studies have shown that the presence of free uronic acids in the pectic-polysaccharides can activate the hydrogen atom of the anomeric carbon (39, 40). However, in this study, the decrease of DE value was mainly attributed to the de-acetylation of galacturonosyl residues in OPP (Figure 2), which might not obviously affect the rate of unmethylated uronic acids. Besides, the antioxidant capacity of pectic-polysaccharides is often closely related to their molecular mass and uronic acids (12, 15, 36, 41). Therefore, in this study, according to the structural differences measured as abovementioned, the slight decrease of antioxidant activity of DOPP-LDE compared to DOPP-HDE might be due to the slight decrease of uronic acid content (Table 1).

### Effects of various degrees of esterification on *in vitro* immunostimulatory activities of okra pectic-polysaccharides

Immunity refers to the protection effects of biological organisms against foreign bacteria, viruses, and other harmful substances. A large number of studies have demonstrated that dietary polysaccharides from edible and medicinal plants can maintain the human health by regulating the immune system (42, 43). Generally, the immunostimulatory effects of dietary polysaccharides are associated with their molecular mass, branched chain length, uronic acid, chain conformation, and glycosidic linkage (44, 45). In fact, several studies have shown that pectic-polysaccharides isolated from different parts of okra possess remarkable *in vitro* and *in vivo* immunostimulatory effects (14, 46–48). A previous study also showed that the immunostimulatory effect of a purified okra pectic-polysaccharide was closely related to the combination effect of molecular mass, branched chain length, and DE (9). However, whether the DE can directly affect the immunostimulatory effect of OPP remains unclear.

**FIGURE 5:**
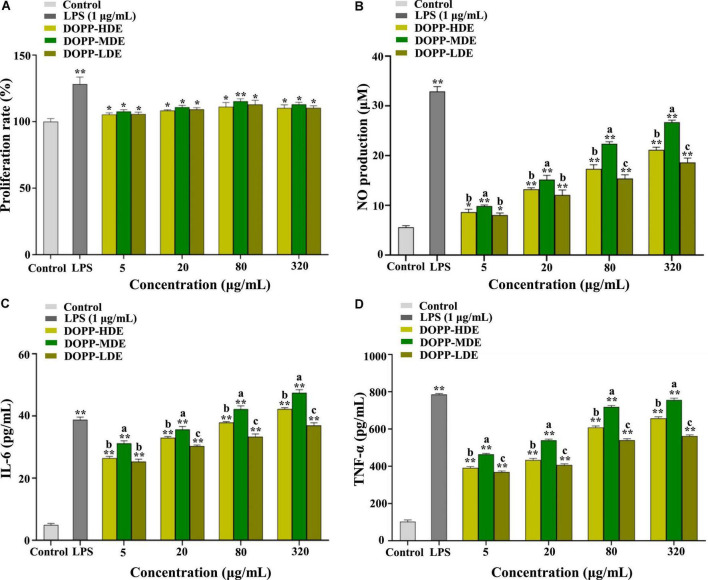
Effects of okra pectic-polysaccharides (OPP) with various degrees of esterification on proliferation **(A)**, nitric oxide (NO) production **(B)**, interleukin-6 (IL-6) production **(C)**, and tumor necrosis factor-α (TNF-α) production **(D)** of RAW 264.7 macrophages. The sample codes were the same in Figure 1; the error bars are standard deviations; significant differences of cell proliferation and release of NO, IL-6, and TNF-α in LPS, DOPP-HDE, DOPP-MDE, and DOPP-LDE vs. control are shown by *p < 0.05, **p < 0.01. Significant differences (*p* < 0.05) of release of NO, IL-6, and TNF-α among DOPP-HDE, DOPP-MDE, and DOPP-LDE are shown by data bearing different letters (a–c).

Therefore, the RAW 264.7 macrophage was applied as a cell model for the determination of immunostimulatory effects of OPP with various DE values. Figure 5 displays the immunostimulatory effects of DOPP-HDE, DOPP-MDE, and DOPP-LDE. As shown in Figure 5A, all tested samples could slightly promote the proliferation of RAW 264.7 macrophages at the concentrations ranged from 5 to 320 μg/mL, indicating that DOPP-HDE, DOPP-MDE, and DOPP-LDE had no toxicity effects. Furthermore, macrophages can exert their functions by secreting NO and various cytokines, such as IL-6 and TNF-α (49). NO is a biologically active cell messenger that plays a critical role in killing pathogenic microorganisms and tumor cells; TNF-α is active in regulating inflammation and autoimmunity; IL is involved in the immune response in the host that plays a key role in maintaining homeostasis. As shown in Figures 5B–D, OPP with different DE values could remarkably promote the release of NO, IL-6, and TNF-α from RAW 264.7 macrophages in a dose-dependent manner, respectively. Interestingly, OPP with various DE values exhibited notably different effects on the release of NO, IL-6, and TNF-α from RAW 264.7 macrophages. More specifically, the higher productions of NO, IL-6, and TNF-α from RAW 264.7 macrophages were found in DOPP-MDE compared to DOPP-HDE, while the lower productions of NO, IL-6, and TNF-α were found in DOPP-LDE compared to DOPP-HDE. Collectively, according to the structural differences among DOPP-HDE, DOPP-MDE, and DOPP-LDE, these results indicated that the immunostimulatory effect of OPP was closely related to its DE. A previous study also showed that the DE played a key role in the immunostimulatory effect of pectic-polysaccharides from *Asparagus officinalis* L., and a relatively high DE value was associated with the relatively high immunostimulatory effect (50). Indeed, the acetyl groups of a purified polysaccharide from *Polygonatum cyrtonema* might also benefit its immunostimulatory effect (51). In addition, several studies have demonstrated that the acetylation modification of natural polysaccharides can enhance their immunostimulatory effects (52, 53), while removing the acetyl groups resulted in the remarkable decrease of immunostimulatory functions (54, 55). Therefore, the findings in the present study indicate that OPP with a DE value of 25.88% possess remarkable *in vitro* immunostimulatory effect, and the complete de-acetylation in DOPP-LDE results in a remarkable reduction of immune response. Nevertheless, the precise structure-immunostimulatory activity relationship of OPP and related mechanism of action are required to be deeply uncovered in the future.

## Conclusion

Pectic-polysaccharides are regarded as one of the most abundant bioactive components in okra. However, the knowledge about the precise structure-bioactivity relationships of OPP is still limited. Therefore, in order to further clarify the potential structure-bioactivity relationship of okra pectic-polysaccharides, effects of various degrees of esterification on *in vitro* antioxidant capacities and immunostimulatory activities of OPP were investigated. Results showed that the decrease of DE was mainly attributed to the de-acetylation of OPP according to the ^1^H NMR spectra analysis. In addition, results showed that the DE value was not related to the antioxidant activity of OPP. However, the immunostimulatory effect of OPP was closely related to its DE value, and a suitable DE value is beneficial to its *in vitro* immunostimulatory effect. Collectively, the findings are beneficial to revealing the effect of esterification degree on antioxidant activity and immunomodulatory activity of OPP. However, due to the limitations of *in vitro* models, it is necessary to evaluate the bioactivities of okra pectic-polysaccharide and its structure dependent relationships in animal models in the future.

## Data availability statement

The original contributions presented in this study are included in the article/supplementary material, further inquiries can be directed to the corresponding authors.

## Author contributions

WL and JL: investigation, formal analysis, resources, software, and writing – original draft. JW: investigation, formal analysis, and validation. YH: investigation and validation. Y-CH: resources and software. D-TW: data curation, methodology, formal analysis, funding acquisition, and writing – review and editing. LZ: methodology, formal analysis, supervision, resources, and writing – review and editing. All authors contributed to the article and approved the submitted version.
